# A retrospective study of open and endoscopic nephron sparing surgery in the treatment of complex renal tumors

**DOI:** 10.12669/pjms.37.4.3457

**Published:** 2021

**Authors:** Ting-ting Li, Jia Feng, Yan-ling Li, Qian Sun

**Affiliations:** 1 Ting-ting Li, Department of Thoracic Surgery, Affiliated Hospital of Hebei University, Baoding 071000, Hebei, China; 2 Jia Feng, Department of Oncology, Affiliated Hospital of Hebei University, Baoding 071000, Hebei, China; 3 Yan-ling Li, Department of Tuberculosis, Affiliated Hospital of Hebei University, Baoding 071000, Hebei, China; 4 Qian Sun Department of General Surgery, Affiliated Hospital of Hebei University, Baoding 071000, Hebei, China

**Keywords:** Nephron-sparing, Complexity, Renal tumours

## Abstract

**Objective:**

To investigate clinical outcomes of open and retroperitoneal laparoscopic nephron-sparing surgery in the treatment of complex renal tumours.

**Methods:**

A retrospective case study was conducted. Patients with complex renal tumours admitted to our hospital between January 2018 and September 2019 were enrolled; the included patients (n=40) were divided into the observation group (open partial nephrectomy, n=20) and control group (laparoscopic partial nephrectomy, n=20) according to operation modes. The operation time, renal warm ischaemia time, intraoperative blood loss, renal pedicle blocking time, intestinal function recovery time, postoperative hospital stay, and postoperative complications were recorded.

**Results:**

Significant differences were noted regarding renal warm ischaemia time, renal pedicle blocking time, intraoperative blood loss, operation time, and postoperative hospital stay between the observation and control groups (*P*<0.05); however, no significant difference was observed in intestinal function recovery time and postoperative drainage days (*P*>0.05).

**Conclusion:**

Open surgery remains the recommended surgical method for the treatment of few complex tumours in the renal hilus region and has gradually become the renal surgery of choice at present, although laparoscopic surgery has evolved tremendously.

## INTRODUCTION

Renal tumours could be further divided into simple and complex renal tumours, according to location, size, number, texture, and adhesion with surrounding tissues. It was believed that early surgical treatment should be performed in patients with operable renal tumours after a definite diagnosis. Partial nephrectomy showed definite clinical effects, with the advantages of sparing nephrons, facilitating renal pedicle vascular control, reducing cardiovascular and cerebrovascular events, and prolonging patient survival.^1^ Moreover, laparoscopic partial nephrectomy had the benefits of inducing less surgical trauma, rapid postoperative recovery, and less abdominal viscera disturbance.^2^ Currently, for complex renal tumours, especially those in the renal hilum area, open surgery remains superior and revealed remarkable advantages over laparoscopic surgery.^3^ In the present study, a clinical analysis was conducted to compare the treatment effects between open partial nephrectomy and laparoscopic partial nephrectomy for complex renal tumours.

## METHODS

This was a retrospective observational study. Patients with complex renal tumours admitted to our hospital between January 2018 and September 2019 were selected; according to operation technique, the patients (n=40) were divided into the observation group (open partial nephrectomy, n=20) and control group (laparoscopic partial nephrectomy, n=20).

### Inclusion Criteria

(1) Patients with a total score > 9 obtained by the Renal nephrometry scoring system^4^ for evaluation (using the corresponding assignments and scoring); (2) patients with renal tumour indicated by preoperative imaging (enhanced CT and MRI) and confirmed renal cell carcinoma by postoperative pathological diagnosis; and (3) renal hilar tumour

The study was approved by the Institutional Ethics Committee of Affiliated Hospital of Hebei University (Approval no. 20180102), and written informed consent was obtained from all the participants.

### Exclusion Criteria

(1) Patients complicated with severe organ lesions (e.g., in the lung, liver), severe cardiovascular and cerebrovascular diseases, multiple tumours, bone metastasis, and other surgical contraindications and (2) patients with a history of abdominal surgery.

The procedures were the same as the standard open partial nephrectomy and retroperitoneal laparoscopic partial nephrectomy. The operation time, renal warm ischaemia time, intraoperative blood loss, renal pedicle blocking time, intestinal function recovery time, postoperative hospital stay, and postoperative complications were recorded.SPSS software version 20.0 was used for data analysis. The measurement data were expressed as mean±standard deviation (x ± s), and student’s t test was applied for comparisons between groups (mainly used for normal distribution data with small sample size (e.g., n<30) and unknown overall standard deviation σ). The enumeration data were expressed as n (%), and χ^2^ test was used for comparisons. *P*<0.05 indicated statistical significance.

## RESULTS

The observation group included 14 males and six females (10 cases on the left and 10 cases on the right), with an average age of 58.5±12.15 years old and average tumour diameter of 6.6±1.9 cm. Conversely, the control group consisted of 12 males and 8 females (11 cases on the left and 9 cases on the right), with an average age of 57.6±11.5 years old and average tumour diameter of 6.8±1.6 cm. No significant difference was noted regarding gender, age, mean diameter, and Renal score between the observation and control groups (Table-I). The operation time, renal warm ischaemia time, and renal pedicle blocking time were significantly shorter in the observation group than in the control group; the observation group had markedly less intraoperative blood loss than the control group; the postoperative drainage volume was evidently less in the observation group than in the control group; and the average postoperative hospital stay of the observation group was obviously longer than that of the control group (all *P*<0.05).

**Table-I:** Comparison of baseline data between control group and observation group (X ± S).

Clinical Parameters	Observation Group (N = 20)	Control Group (N = 20)	P
Age (Year)	58.5 ± 12.15	57.6 ± 11.5	> 0.05
***Gender (N, %)***			
Male	14 (70)	12 (60)	> 0.05
Female	6 (30)	8 (40)	> 0.05
Maximum Diameter of Tumor (Cm)	6.6 ± 1.9	6.8 ± 1.6	> 0.05
***Tumor Site (N, %)***			
Left Side	10 (50%)	11 (55%)	> 0.05
Right Side	10 (50%)	9 (45%)	> 0.05
Upper Pole	10 (50%)	9 (45%)	> 0.05
Middle Pole	4 (20%)	5 (25%)	> 0.05
Lower Pole	6 (30%)	6 (30%)	> 0.05

No significant difference was observed in the intestinal function recovery time and the postoperative drainage time between the observation and control groups (all *P*>0.05) (Table-II).

**Table-II:** Comparison of Perioperative Data between Control Group and Observation Group (X ± S).

Clinical Parameters	Observation Group (N = 20)	Control Group (N = 20)	*T*	*P*
Operation Time (Min)	121.9 ± 10.2	135.1 ± 12.2	2.62	< 0.05
Renal Warm Ischemia Time (Min)	15.2 ± 4.3	23.1 ± 5.2	3.71	< 0.05
Renal Pedicle Blocking Time (Min)	20.6 ± 5.7	29.8 ± 5.1	3.81	< 0.05
Intraoperative Blood Loss (Ml)	125.2 ± 45.5	162.3 ± 30.6	2.13	< 0.05
Postoperative Hospital Stay (D)	12.8 ± 1.5	10.2 ± 2.1	3.25	< 0.05
Postoperative Drainage Volume (Ml)	236.7 ± 10.56	250.6 ± 15.13	2.38	< 0.05
Postoperative Intestinal Function Recovery Time (H)	20.3 ± 4.5	18.5 ± 3.5	1	> 0.05
Postoperative Drainage Time (D)	4.3 ± 0.3	3.9 ± 0.6	1.9	> 0.05

## DISCUSSION

Laparoscopic technology has developed and has shown notable advantages over conventional open surgery, particularly transperitoneal and retroperitoneal surgery.^5^ For partial nephrectomy, the endoscopic technique was completely adequate for simple excision of renal tumours.^6^ Laparoscopic surgery has gradually become a routine operation popular among modern surgeons because of its advantages, such as short operation time, less trauma to patients, fast postoperative recovery, short hospital stay, and appreciable incision aesthetic, among others.^7^-^10^ However, for some complex renal tumours, especially for tumours in the renal hilus region, the laparoscopic technique may induce adhesion problems due to the uncommon location (extremely close to the renal pedicle).^11^ Additionally, it was difficult to identify the junctional zone between the tumour and renal pedicle or normal tissue under laparoscopy, which may result in increased difficulty of operation, increased risk of operation, difficulty in controlling operation outcome and operation time, and the possibility of intraoperative conversion to open surgery; thus, a psychological test was conducted among the operators and patients.^12^ Moreover, laparoscopic surgery had limitations in implementation for huge pyelogenic cysts, huge renal angiomyolipoma, and duplication of kidney and tumours with multiple calcification due to great difficulty.

Several scholars believed that the tumour around the renal hilum was not the absolute contraindication for laparoscopic surgery hence, the application of laparoscopic surgery still needed to be considered objectively. Before the upsurge of endoscopic technique, open surgery was the only choice of surgeons for the excision of diseased tissues.^13^ It was reported that open surgery was extremely helpful for partial nephrectomy and has been applied for a long time. Furthermore, open surgery had more significant advantages for tumours in the renal hilus region.^14^ Firstly, open surgery could be performed under direct viewing, fully mobilising the surgeon’s senses and making it convenient and quick to separate and block the renal pedicle and identify the tumour junctional zone, shortening the operation time. Secondly, open operation could greatly shorten lesion resection time, renal pedicle vascular occlusion time, and warm ischaemia time and protects the nephron function better.^15^ Moreover, with the complexity in tumour location and size, the possibility of intraoperative emergencies increased. Open surgery could timely and effectively manage emergencies, which was not possible in the laparoscopic technique.^16^ Compared with laparoscopic surgery, open surgery had some disadvantages, such as unattractive incision, increased postoperative pain, and relatively long hospital stay, among others. It was demonstrated that open surgery and laparoscopic surgery in partial nephrectomy had various advantages and disadvantages.^17^

In the present study, the clinical effects of open surgery and laparoscopic surgery were compared in the treatment of complex tumours in the renal hilus region. Open surgery showed more advantages than laparoscopic surgery in terms of operation time, renal warm ischaemia time, renal pedicle blocking time, and intraoperative blood loss and postoperative drainage volume. However, no significant difference was noted regarding intestinal function recovery time and postoperative drainage day between open surgery and laparoscopic surgery. Additionally, no postoperative complication was found in the observation and control groups. These findings suggested that open surgery had some advantages for patients with tumours in the renal hilus region undergoing partial nephrectomy, wherein advantages of open surgery were mainly reflected in the following aspects: first, open surgery involved multiple issues, including vision, touch, and hearing, making the operation faster and anatomical level clearer, protecting the renal pedicle to the maximum extent, reducing the incidence of positive surgical margins, and reducing the possible postoperative recurrence; second, the sutures of the collecting system and renal parenchyma under open and direct vision were more firm and accurate, thus greatly reducing the incidence of postoperative complications; third, ice water cooling was applied around the kidney according to the condition, to increase the tolerance of kidney tissues to ischaemia and hypoxia^18^ and retain the function of residual nephrons to the maximum extent; and last, open surgery could easily manage emergencies caused by the difficulty of operation and could block blood vessels and stop bleeding promptly, if necessary. Furthermore, radical nephrectomy can save the lives of patients to the maximum degree.^19^,^20^ This study showed that although laparoscopic partial nephrectomy is widely used, open surgery plays a crucial role.

### Limitations of the Study

First, this was not a prospective randomised controlled study. Second, the popular Da Vinci robot-assisted laparoscopic partial nephrectomy was not included in the comparative study.

## CONCLUSION

Open surgery remains the recommended surgical method for the treatment of a few complex tumours in the renal hilus region and has gradually become the renal surgery of choice, although laparoscopic surgery has developed a lot.

### Authors’ Contributions:

**JF** & **TTL:** designed this study and prepared this manuscript, and are responsible and accountable for the accuracy or integrity of the work.

**QS:** collected and analyzed clinical data.

**YLL:** significantly revised this manuscript.

**TTL** & **JF:** are both considered as first author.
